# SNARE Zippering and Synaptic Strength

**DOI:** 10.1371/journal.pone.0095130

**Published:** 2014-04-18

**Authors:** Rene C. Prashad, Milton P. Charlton

**Affiliations:** Department of Physiology, University of Toronto, Toronto, Ontario, Canada; University of Edinburgh, United Kingdom

## Abstract

Synapses vary widely in the probability of neurotransmitter release. We tested the hypothesis that the zippered state of the *trans*-SNARE (Soluble N-ethylmaleimide-sensitive factor Attachment protein REceptor) complex determines initial release probability. We tested this hypothesis at phasic and tonic synapses which differ by 100-1000-fold in neurotransmitter release probability. We injected, presynaptically, three *Clostridial* neurotoxins which bind and cleave at different sites on VAMP to determine whether these sites were occluded by the zippering of the SNARE complex or open to proteolytic attack. Under low stimulation conditions, the catalytic light-chain fragment of botulinum B (BoNT/B-LC) inhibited evoked release at both phasic and tonic synapses and cleaved VAMP; however, neither BoNT/D-LC nor tetanus neurotoxin (TeNT-LC) were effective in these conditions. The susceptibility of VAMP to only BoNT/B-LC indicated that SNARE complexes at both phasic and tonic synapses were partially zippered only at the N-terminal end to approximately the zero-layer with the C-terminal end exposed under resting state. Therefore, the existence of the same partially zippered state of the *trans*-SNARE complex at both phasic and tonic synapses indicates that release probability is not determined solely by the zippered state of the *trans*-SNARE complex at least to the zero-layer.

## Introduction

The probability that a presynaptic action potential will cause release of a synaptic vesicle varies greatly between synapses (reviewed in [1]). For instance, at cerebellar Purkinje cells, synapses made by climbing fibers have a far higher probability than those made by granule cells [2] (reviewed in [3]). The mechanism of this presynaptic differentiation is largely unknown.

**Figure 1:**
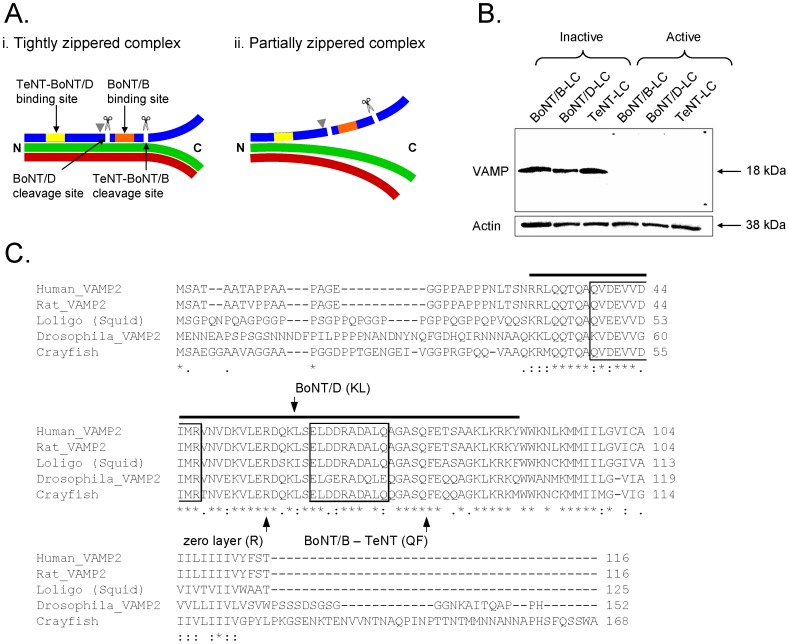
Susceptibility of VAMP to *Clostridial* neurotoxins. ***A***, Zippered states of the SNARE complex. ***Ai***, SNARE complex tightly zippered beyond the zero-layer indicated by gray arrow head. VAMP (blue) is protected from cleavage because the binding (yellow and orange bars) and cleavage (scissors and white lines) sites of VAMP-specific *Clostridial* neurotoxins (TeNT, BoNT/B and BoNT/D) are occluded. ***Aii***, Partially zippered SNARE complex. The binding sites of TeNT and BoNT/D are occluded but the binding and cleavage sites for BoNT/B are exposed such that VAMP is susceptible to cleavage. Green – syntaxin, red – SNAP25 (represents both SNARE binding motifs). ***B***, *Clostridial* neurotoxins cleave crayfish VAMP *in-vitro*. Crayfish CNS protein sample was incubated with inactive or active neurotoxins (BoNT/B-LC (0.5 µg/μL), BoNT/D-LC (0.3 µg/μL) and TeNT-LC (0.5 µg/μL)) and stained for neuronal VAMP. The protein bands of 18 kDa represent VAMP. Cleaved VAMP does not appear on the blot when active neurotoxins were used because the VAMP antibody binds only to the uncleaved VAMP protein. Actin staining of 38 kDa below the VAMP blot shows that equal amounts (10 µg) of protein were loaded in each lane. ***C***, Comparison of full-length crayfish VAMP amino acid sequence with VAMP sequences from other species. Crayfish VAMP is similar to VAMP from other species, especially in the conserved SNARE motif region (black bar). The cleavage sites of VAMP-specific neurotoxins are indicated in the alignment. The primary binding sites of the neurotoxins used in this study are indicated as boxed regions (V1 motif (aa 38–47) – TeNT and BoNT/D; V2 motif (aa 62–71) – BoNT/B) based on the human VAMP sequence [29]–[31]. The multiple protein sequence alignment was performed using the online ClustalW2 Multiple Sequence Alignment tool (European Molecular Biology Laboratory - European Bioinformatics Institute, http://www.ebi.ac.uk/Tools/msa/clustalw2/).

The Ca^2+^-dependent process of vesicle fusion requires the interaction between the three SNARE proteins: syntaxin, SNAP-25, and VAMP. The SNAREs interact in a process known as zippering, which occurs initially at their N-termini and proceeds in the C-terminal direction, forming a tight *trans*-SNARE complex (Fig. 1*A*) that triggers vesicle fusion [4]–[8]. The SNARE complex can exist in either a tightly or partially zippered state in which only the tightly zippered state is fusogenic. The partially zippered complex is observed in various cell systems or *in-vitro* assays [6], [9]–[13]. There is evidence to show that the SNARE complex can fluctuate between different zippered states [9], [13]. Furthermore, a mutation of syntaxin that promotes formation of the SNARE complex causes increased neurotransmitter (NT) release at *Drosophila* neuromuscular junctions [14]. Therefore, different zippered states might contribute to differences in the initial release probability at synapses: where SNAREs are less zippered, more steps would be required to reach the fully zippered state needed for vesicle fusion and probability of release would be reduced.

We inferred the zippered state of SNARE complexes at synapses from the differential proteolytic activity of presynaptically injected *Clostridial* neurotoxins. Previous studies have shown that SNARE-specific neurotoxins can help to determine the zippered state of the SNARE complex because neurotoxins are effective only when both their binding and cleavage sites on SNAREs are exposed or unzippered [9], [15]–[19] (Fig. 1*A*). In this study, we used neurotoxins that specifically target VAMP: two of the neurotoxins cleave only unzippered VAMP while the third can cleave partially zippered VAMP [15], [20].

We investigated presynaptic strength differentiation at the crayfish walking leg extensor muscle where phasic synapses have an initial release probability that is 100-1000-fold higher with a Ca^2+^ sensitivity of release that is 10-fold greater than tonic synapses [21], [22]. The phasic synapses exhibit depression of NT release with high frequency stimulation whereas the tonic synapses facilitate [22]–[24]. The phasic synapses have a smaller ready-releasable pool (RRP) size [24], lower number and oxidative activity of mitochondria [25], [26], and higher [Ca^2+^]_i_ during a single action potential [27] than the tonic synapses; these differences cannot account for the higher release probability of phasic synapses [22], [24]. Therefore, we ask whether differences in the probability of NT release are due to differences in SNARE zippering. More specifically, we hypothesized that *trans*-SNARE complexes associated with fusion-competent vesicles are zippered past the zero-layer at high probability phasic synapses, but zippered no further than the zero-layer at low probability tonic synapses (see Fig. 1*A*).

## Materials and Methods

### Animals and preparation

Crayfish (*Procambarus clarkii*, 5–6.5 cm long) were purchased from Atchafalaya Biological Supply Company (Dantin, LA) and housed in a tank filled with aerated, de-chlorinated tap water at a temperature of 12–14°C under an 8–16 hrs light-dark cycle. The water was changed once a week and the crayfish were fed lentils. The first or second walking leg was removed from the cephalothorax (basipodite region) of the animal by autotomy. The leg was pinned down dorsal side up in a Sylgard-lined 35 mm Petri dish filled with a modified Van Harreveld crayfish saline solution [28] containing the following (in mM): 205.3 NaCl, 5.40 KCl, 13.5 CaCl_2_·2H_2_O, 2.70 MgCl_2_·6H_2_O, and 10 N-[2-Hydroxyethyl] piperazine-N′-[2-ethanesulfonic acid] (HEPES) dissolved in distilled water (dH_2_O) and titrated to a pH of 7.40 using 1 N NaOH_(aq)_. The osmolality of the crayfish saline was 410–430 mOsm. Incisions were made along the lateral sides of the meropodite region of the leg to remove the dorsal cuticle and underlying flexor muscle and expose the extensor muscle. The main nerve bundle was removed to expose the phasic and tonic axons, which can be distinguished morphologically by their position and diameter. Features of the extensor muscle preparation have been described previously [23]. The muscle was stretched to minimize muscle contractions during electrophysiological stimulation by cutting the membrane that connects the meropodite and carpopodite regions but leaving the tendon intact, which was pulled and kept taught using insect pins. Experiments were performed at room temperature (22°C).

### Electrophysiological recordings

To determine the amount of NT release, the phasic or tonic axon was stimulated and the corresponding excitatory postsynaptic potential (EPSP) was recorded from the extensor muscle using an intracellular sharp, 1.5 mm thick-walled borosilicate glass micropipette (10–15 MΩ, 3 M KCl; Sutter Instrument Co., Novato, CA) pulled on a Flaming/Brown micropipette puller (model P-97, Sutter Instrument Co.). The phasic axon was stimulated using a cuff electrode connected to an external stimulator (Model 2100, Isolated Pulse Stimulator, A-M Systems, Carlsborg, WA) and triggered by the WINWCP software (ver. 4.0.8, written by John Dempster, University of Strathclyde, Glasgow, Scotland). The tonic axon was stimulated by passing a suprathreshold current via an amplifier (Intracellular Electrometer IE-201, Warner Instruments Corp., Hamden, CT) through a sharp glass micropipette (10–15 MΩ, 3 M KCl), which was used to impale the primary branch of the tonic axon, and triggered using the WINWCP software.

#### Low frequency stimulation

**Figure 2:**
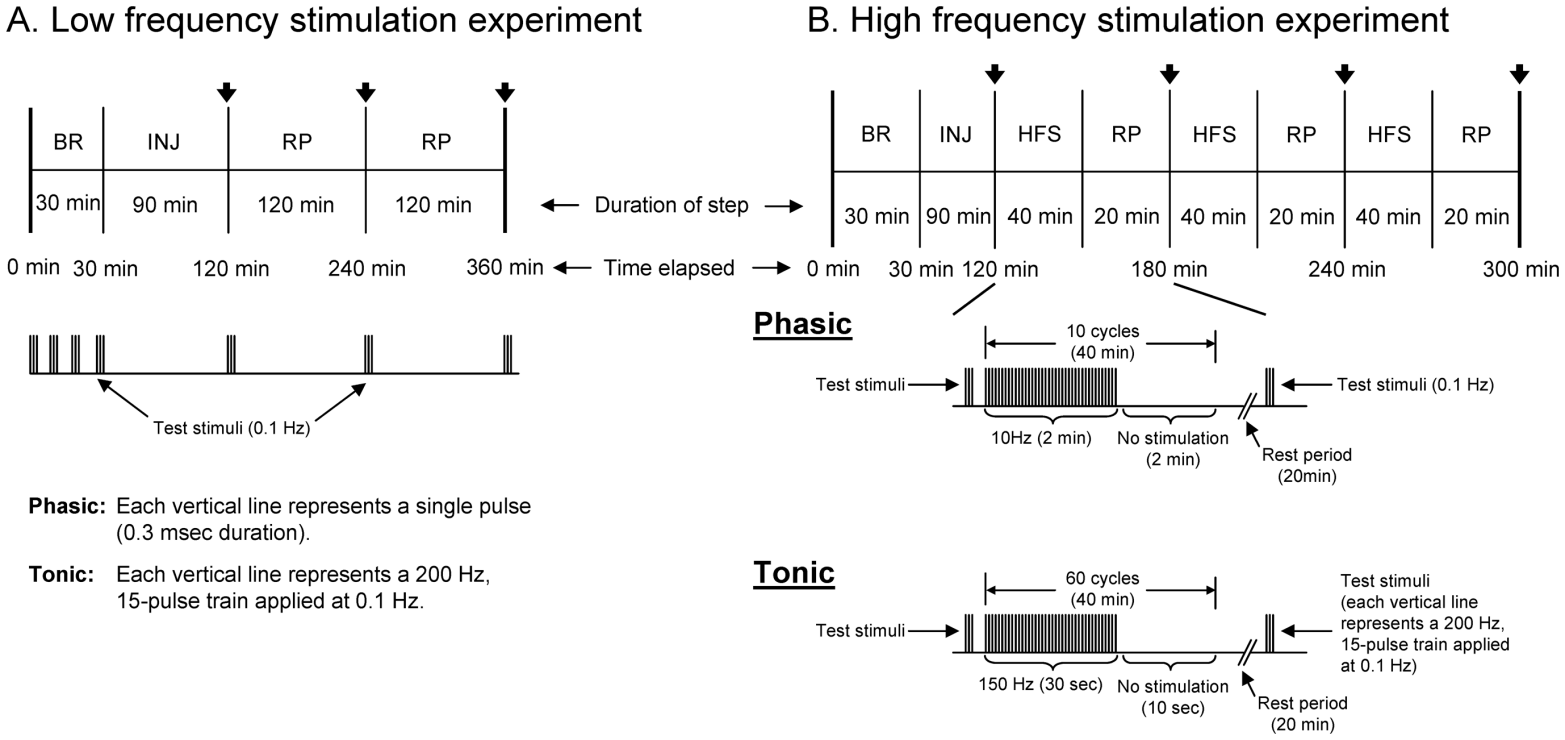
Timeline of the physiological recordings for neurotoxin injection experiments. ***A***, Timeline of the low frequency stimulation experiment. ***B***, Timeline of the high frequency stimulation experiment. In *A* and *B*, the black arrows (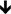) mark the time when test stimuli (Phasic – three single pulses at 0.1 Hz; tonic – three, 200 Hz, 15- pulse trains at 0.1 Hz) were applied following injection, and below each timeline is a diagram depicting the stimulation protocol for phasic and tonic recordings (in *B*, an example of the high frequency stimulation (HFS) protocol is given from time elapsed mark of 120 min to 180 min). Baseline recording (BR): Phasic – three single pulses at 0.1 Hz every 10 min; tonic – three, 200 Hz, 15-pulse trains at 0.1 Hz every 10 min. High frequency stimulation (HFS): Phasic – burst stimuli of 10 Hz for 2 min with an inter-burst interval of 2 min; tonic – burst stimuli of 150 Hz for 30 sec with an inter-burst interval of 10 sec. INJ – injection, RP – rest period.

To determine the effects of the *Clostridial* neurotoxins under conditions close to resting state, a low frequency stimulation (LFS) paradigm was used to evoke NT release using as few stimuli as possible. Baseline recordings were taken every 10 min for 30 min followed by pressure injection of one of the three neurotoxins. At each time point, the phasic axon was stimulated using a single square wave stimulus (0.3 msec duration) of suprathreshold amplitude to evoke a response, whereas the tonic axon was stimulated using a train of 15 square wave stimuli (each 0.3 msec in duration) delivered at 200 Hz. A train of stimuli rather than a single pulse was needed to generate tonic EPSPs because a single pulse was not sufficient to evoke a tonic EPSP due to the low probability of release at tonic synapses [22]–[24]. After neurotoxin injection, phasic or tonic responses were recorded immediately, 2 hrs and 4 hrs after injection to determine the effect of the neurotoxin on the evoked response. The phasic axon was stimulated using the phasic baseline stimulation protocol and the average of three EPSPs at each time point was used for analyses. The tonic axon was stimulated using the tonic baseline stimulation protocol and the average of three EPSPs (last EPSP in each trace) was used at each time point for analyses. The measured responses were normalized by expressing each measured EPSP amplitude as a percentage of the initial baseline EPSP amplitude (time  =  0 min). The timeline of this protocol is given in Figure 2*A*.

#### High frequency stimulation

To test the effects of the *Clostridial* neurotoxins under conditions mimicking high synaptic activity, a high frequency stimulation (HFS) paradigm was used. Baseline recording was the same as the low frequency stimulation protocol; however, after neurotoxin injection the phasic axon was stimulated using a burst of 10 Hz for 2 min with an inter-burst interval of 2 min for a total of 40 min. The tonic axon was stimulated using a burst of 150 Hz for 30 sec with an inter-burst interval of 10 sec for a total of 40 min. The inter-burst interval in which no stimulation was applied was necessary to allow the axon to recover otherwise conduction failure would occur and to prevent stimulation-induced depression which readily occurs at phasic synapses. The primary goal was to evoke as much stimulation as possible to cause rapid turnover of SNARE complexes. After each 40 min round of stimulation, a rest period of 20 min was given prior to taking a test response. This stimulation paradigm was repeated two more times for a total elapsed time of 3 hrs. The timeline of this protocol is given in Figure 2*B*.

The 20 min rest period following each round of stimulation was necessary because the tonic response, and more so, the phasic response showed signs of potentiation such that without the rest period the response would be much larger than the initial baseline recordings. Even with the 20 min rest period, however, there were instances in which the phasic test response was larger than the initial baseline recordings. The goal was to minimize the potentiated effect of the evoked response due to high frequency stimulation which could mask the effects of the neurotoxins; however, synaptic potentiation could not be completely avoided, especially for the phasic response. The overall goal was to establish if the neurotoxins have a stimulation-dependent effect at the synapses.

#### Action potential recordings

Phasic and tonic axon action potentials were recorded using an intracellular sharp, thick-walled glass micropipette (10–15 MΩ, 3 M KCl) as each axon was stimulated using the cuff electrode to apply a single square wave stimulus (0.3 msec duration) of suprathreshold amplitude at 0.1 Hz. The average amplitude of three action potentials was taken after baseline EPSP recordings and at the end of the experiment following the last EPSP recording.

### Data collection and analysis

Analog signals were low pass filtered at 2 kHz using a 4-pole Bessel filter, amplified 10-fold by a model LPF202 amplifier (Warner Instruments Corp.) and digitized at 10 kHz (Axon Digidata 1200, Molecular Devices. Inc., Sunnyvale, CA) under control of the WINWCP software.

The peak amplitude of the phasic and tonic EPSPs was used as a measure of the amount of NT released. The amplitude of the phasic EPSP was measured as the average of three EPSP responses taken at each time point at 0.1 Hz. To measure the peak amplitude of the tonic response at each time point, first the peak amplitude of the last EPSP (corresponding to the 15^th^ pulse in the 200 Hz train) of three traces (0.1 Hz apart) was measured separately and then the values were averaged. This yielded the average peak amplitude of the last EPSP in the tonic response. The Axon pClamp software (ver. 9.0, Molecular Devices Inc.) was used to analyze evoked phasic and tonic responses. The amplitude of the EPSP at each time point was normalized as a percentage of the initial baseline EPSP amplitude at time  =  0 min. Testing for statistical difference between two groups was achieved using the Student's t-test. A statistical difference was achieved when p<0.05. All data presented represent the average response using a sample size (n) of five or greater.

### Pressure injection

The tip of a sharp glass micropipette was filled with protein solution and then backfilled with 100 mM KCl (40–55 MΩ). Once an axon was impaled with the micropipette, a Picospritzer II microinjector (General Valve Corporation, Pine Brook, NJ) was used to inject the protein solution into the axon using a pressure of 172–275 kPa (25–40 psi) with the duration of each air pulse at 5–30 msec applied every 10 sec for 90 min. Evoked EPSP recordings were not made during injection because muscle contractions would dislodge the injection micropipette or movement of the axon impaled with the micropipette would cause axonal damage. Stretching the extensor muscle was not sufficient to completely inhibit muscle contraction.

### Western blotting

The VAMP antibody was tested for specificity by Western blotting of proteins extracted from crayfish nerve cords. In addition, the specificity and activity of *Clostridial* neurotoxins against VAMP were tested. Proteins were extracted by freezing 20 crayfish nerve cords on dry ice and homogenizing the tissue sample in a protein extraction buffer containing: 50 mM Tris-HCl, 150 mM NaCl, 10 mM dithiothreitol (DTT), 1% (w/v) sodium deoxycholate, 1% (v/v) Triton X-100 and a protease inhibitor cocktail (Complete Mini EDTA-Free Protease Inhibitor Cocktail, Roche Diagnostics, Laval, QC, Canada). Next, the protein sample was placed in a boiling water bath for 10 min and then centrifuged at 12,000×g for 10 min, after which the supernatant was collected. The protein sample concentration was at least 1.0 µg/μL, which was measured using the Protein dotMETRIC Assay kit (G-Biosciences, Maryland Heights, MO). The protein sample was subjected to SDS-PAGE on a Ready Gel precast polyacrylamide 4–15% Tris-HCl gradient gel (Bio-Rad, Hercules, CA) using the Mini-Protean III electrophoresis unit (Bio-Rad) and transferred to a nitrocellulose membrane using the Mini PROTEAN III Trans-Blot system (Bio-Rad). After incubating the membrane in blocking solution (100 mM Tris-HCl, 154 mM NaCl, 0.1% (v/v) Tween-20, 5% (w/v) powdered skimmed milk and 2% BSA, pH = 7.4) for 2.5 hrs at 22°C, the blot was probed with a 1∶500 dilution of a guinea pig polyclonal VAMP antibody (made to the SNARE motif of human VAMP-2, amino acids 33–94; gift from Dr. C.C. Shone, University of Bath, Claverton Down, Bath, UK) and a 1∶1000 dilution of a rabbit polyclonal actin antibody (used as a loading control; A5060, Sigma-Aldrich, Oakville, ON, Canada) overnight at 4°C. Then, the blot was probed with a 1∶2000 dilution of a goat anti-guinea pig IgG tagged with HRP (Jackson ImmunoResearch Laboratories, Inc., West Grove, PA) and a 1∶2000 dilution of a goat anti-rabbit IgG tagged with HRP (Jackson ImmunoResearch Laboratories, Inc.) for 2.5 hrs at 22°C. Protein bands were detected using a chemiluminescence solution (NEL105001EA, Western Lightning Plus-ECL, Perkin Elmer, Waltham, MA) and then imaged using the Kodak Image Station 2000R (Mandel Scientific Company Inc., Guelph, ON, Canada).

### Immunocytochemistry

The dissected meropodite region of the crayfish 1^st^ or 2^nd^ walking leg was pinned down in a Sylgard-lined Petri dish with the leg extensor muscle stretched using insect pins to minimize distortion and shrinkage during fixation. The preparations were first fixed in phosphate buffer solution (PBS: 10 mM Na_2_HPO_4_, 2 mM KH_2_PO_4_, 140 mM NaCl and 2.7 mM KCl, pH 7.40, 410–430 mOsm) with 4% (v/v) paraformaldehyde (Polysciences, Inc., Warrington, PA) for 1 hr at 22°C. Then, the preparations were washed in PBS for 15 min on an orbital shaker with three changes of PBS. Next, the preparations were placed in blocking solution (PBS containing 0.1% (v/v) Triton X-100 and 1% (w/v) BSA) for 1.5 hrs at 22°C on an orbital shaker to block non-specific antibody binding. Then, the preparations were incubated overnight at 4°C on an orbital shaker in the primary antibody solution containing: PBS, 0.1% (v/v) Triton X-100, 1∶100 dilution of the guinea pig polyclonal VAMP antibody and a 1∶300 dilution of a rabbit polyclonal synapsin antibody (gift from Dr. H-T. Kao, Brown University, Providence, RI). The next day, the preparations were washed in PBS-T (PBS containing 0.1% (v/v) Triton X-100) for 25 min at 22°C on an orbital shaker with the solution changed five times. Then, the preparations were incubated for 2.5 hrs at 22°C on an orbital shaker in the secondary antibody solution containing: PBS, 0.1% (v/v) Triton X-100, 1∶500 dilution of a goat anti-guinea pig IgG (H+L) tagged with Alexa 488 fluorescent dye (Life Technologies Corp., Carlsbad, CA) and 1∶500 dilution of a goat anti-rabbit IgG (H+L) tagged with Alexa 594 fluorescent dye (Life Technologies Corp.). Finally, the preparations were washed in PBS-T for 25 min at 22°C on an orbital shaker with the solution changed five times. The preparations were imaged using a Leica TCS SL laser confocal microscope (software version 2.61, build 1537 181.031, Leica Microsystems, Wetzlar, Germany). Images were taken using a 40× (N.A. 0.80) or a 63× (N.A. 1.20) water immersion objective, and 488 nm and 543 nm excitation wavelengths. Images from different focal planes were stacked together to produce a projected image.

### Clostridial neurotoxin preparation for Western blotting

VAMP-specific tetanus neurotoxin light-chain (TeNT-LC) and botulinum neurotoxins B and D light-chain (BoNT/B-LC and BoNT/D-LC, respectively) were purchased from List Biologicals Laboratories, Inc. (Campbell, CA). Control neurotoxin solutions were placed in a boiling water bath for 30 min to denature and inactivate the neurotoxins.

### Clostridial neurotoxin preparation for pressure injection

The lyophilized form of TeNT-LC and BoNT/B/D-LC was reconstituted with a solution consisting of 100 mM KCl and 300 µM 3 kDa neutral dextran-Texas red (Life Technologies Corp., Carlsbad, CA) such that the concentration of TeNT-LC and BoNT/B-LC were 0.5 µg/μL and BoNT/D-LC was 0.3 µg/μL. The fluorescent dextran-Texas red dye was used to allow visualization of neurotoxin injection. Control neurotoxin solutions were denatured in a boiling water bath for 30 min.

### Cloning and sequencing crayfish VAMP

#### Total RNA extraction and cDNA production

Total RNA was obtained by freezing on dry ice and mechanically homogenizing 20 crayfish nerve cords. TRI-Reagent (Sigma-Aldrich) was applied to the homogenized sample to extract total RNA as per the manufacturer's instructions. The extracted RNA sample was subjected to reverse transcription to generate cDNAs using SuperScript III Reverse Transcriptase (#18080-093, Life Technologies Corp.) as per the manufacturer's instructions.

#### Crayfish VAMP SNARE motif sequence

The Fermentas GeneJet Fast 2× Master Mix kit (# K0171, Fermentas Canada Inc., Burlington, ON, Canada) was used to prepare the cDNA sample for PCR, in which 10 µM of the forward primer (5′- GGTGGATGAGGTGGTGGACATCATGAG -3′) and reverse primer mixture (5′- ATGATCATCATCTTGCAGTTYTTCCACC -3′) were added. Amplification was achieved using hot-start, touchdown PCR with 30 cycles to yield a PCR product of approximately 180 bp. The PCR product was gel purified and ligated to a TA vector using the Qiagen Cloning Kit (# 231122, Qiagen, Germantown, MD) and then amplified in Stratagene XL1-Blue Subcloning-Grade competent bacterial cells (# 200130, Aligent Technologies Inc., Santa Clara, CA). The recombinant TA vectors were extracted and purified using the Qiagen Qiaprep Spin Miniprep kit (# 27106, Qiagen) and submitted for sequencing at The Centre for Applied Genomics (The Hospital for Sick Children, MaRS Centre, Toronto, ON, Canada).

#### Full-length crayfish VAMP sequence

The full-length crayfish VAMP sequence was determined using 5′- and 3′- RACE procedures using the Clontech SMART RACE Amplification Kit (# 634914, Clontech Laboratories Inc., Mountain View, CA) as per the manufacturer's instructions.

#### 5′- and 3′- RACE

The 5′-RACE procedure was employed to determine the VAMP mRNA sequence that corresponds to the protein region N-terminal to the SNARE motif. Amplification was achieved using hot-start, touchdown PCR with 35 cycles using 10 µM of the Clontech Nested Universal Primer A (5′- AAGCAGTGGTATCAACGCAGAGT-3′) supplied with the SMART RACE kit, and the nested VAMP-specific reverse primer (5′- CCACCACATTTTCCTCTTTAGTTTG -3′). The 3′-RACE procedure was employed to determine the VAMP mRNA sequence that corresponds to the protein region C-terminal to the SNARE motif. Amplification was achieved using hot-start, touchdown PCR with 35 cycles using 10 µM of the nested VAMP-specific forward primer (5′-GTGCTCGAGAGAGATCAGAAACTC -3′) and the Clontech Nested Universal Primer A (5′- AAGCAGTGGTATCAACGCAGAGT-3′) supplied with the SMART RACE kit. Similar to the VAMP SNARE motif PCR product, the RACE PCR products were gel purified, amplified and submitted for sequencing. The full-length crayfish VAMP sequence was determined by combining the SNARE motif sequence with the 5′- and 3′- RACE sequences. The full-length VAMP sequence is given in Figure 1*C* (GenBank accession number KF773142, GI: 575488803).

## Results

### Crayfish neuronal VAMP contains the conserved binding and cleavage sites of *Clostridial* neurotoxins

Our experimental strategy was to detect differential effects of TeNT-LC, BoNT/B-LC and BoNT/D-LC as an indicator of differential SNARE zippering at phasic and tonic synapses. Therefore, we determined the sequence of crayfish neuronal VAMP to confirm that it contained the binding and cleavage sites of the *Clostridial* neurotoxins. The sequence in Figure 1*C* shows that crayfish neuronal VAMP contains the binding and cleavage sites of the three neurotoxins used in this study (TeNT, BoNT/B and BoNT/D) in addition to those of BoNT/F but does not contain the cleavage site for BoNT/G [29]–[33]. Therefore, crayfish neuronal VAMP is susceptible to TeNT-LC, BoNT/B-LC and BoNT/D-LC. Furthermore, the sequence alignment in Figure 1*C* shows that the crayfish VAMP amino acid sequence is homologous to VAMP from other species, and the SNARE motif region that binds with the other two SNARE proteins to form the SNARE complex is conserved amongst the sequences.

### Cleavage of VAMP by *Clostridial* neurotoxins *in-vitro*

To verify the activity and specificity of the light-chain form of *Clostridial* neurotoxins responsible for binding to and cleaving VAMP (TeNT-LC, BoNT/B-LC and BoNT/D-LC), crayfish nerve cord protein samples (10 µg) were mixed with one of the three neurotoxins solutions (active or inactive forms, see *Materials and Methods*) in a 1.5 mL microfuge vial and placed in an incubator-shaker at 37°C, 170 rpm for 2.5 hrs. After incubation, the samples were subjected to SDS-PAGE and Western blotting using a VAMP antibody that only binds to the uncleaved form of crayfish VAMP [6], [20], [34]–[36] and an actin antibody to detect actin, which was used as a measure of equal loading of protein samples across all lanes (actin is not susceptible to the neurotoxins) (see *Materials and Methods* for details).

The three neurotoxins used in this study are of the same serotypes used by [6], [20] but were obtained from a commercial source. Therefore, we wanted to determine if the neurotoxins used in this study would produce effects similar to those produced by the neurotoxins used previously. The Western blot (Fig. 1*B*) showed that inactivated neurotoxins did not cleave crayfish VAMP because a single band representing VAMP of 18 kDa for each control sample was observed. In the presence of each active neurotoxin, however, VAMP staining was not observed. This indicated that VAMP was cleaved by each neurotoxin *in-vitro*, supporting the finding that crayfish VAMP contains the binding and cleavage sites for BoNT/B, BoNT/D and TeNT. The VAMP antibody revealed a single band for each control neurotoxin solution. It would appear that there is only one isoform of crayfish neuronal VAMP, which would parallel the cloning and sequencing results for crayfish VAMP showing the presence of only one amino acid sequence (see Fig. 1*C*). The presence of another VAMP isoform of similar molecular weight, however, cannot be ruled out. For instance, VAMP-1 and -2 isoforms can have similar molecular weights and therefore can appear in approximately the same spot on a Western blot [37]–[39], or the VAMP antibody may detect only one of multiple isoforms that might be expressed in the crayfish nervous system.

### The SNARE complex is partially zippered at rest at both phasic and tonic synapses

The muscle fibers of the extensor muscle are innervated by two axons one of which makes phasic synapses while the other makes tonic synapses. The axons can be distinguished morphologically by their position and diameter, and the boutons are larger for tonic axonal terminals compared to phasic axonal terminals. The large diameter of these axons permits intracellular injection of large molecules. To determine the zippered state of the *trans*-SNARE complex under resting state at phasic and tonic synapses, TeNT-LC, BoNT/B-LC and BoNT/D-LC were injected into the phasic and tonic axons. To evaluate the zippered state of the *trans*-SNARE complex we asked whether the synaptic response changed under low frequency stimulation (Fig. 2*A*). The low frequency stimulation (LFS) paradigm was designed to apply as few stimuli as possible to assay NT release under conditions close to resting state. The phasic response exhibited depression that is superimposed on all phasic responses. To help eliminate the effects of this depression for analyses, the percent difference between active and inactive neurotoxin was measured, which would reveal the true effect of each neurotoxin on the evoked response.

**Figure 3:**
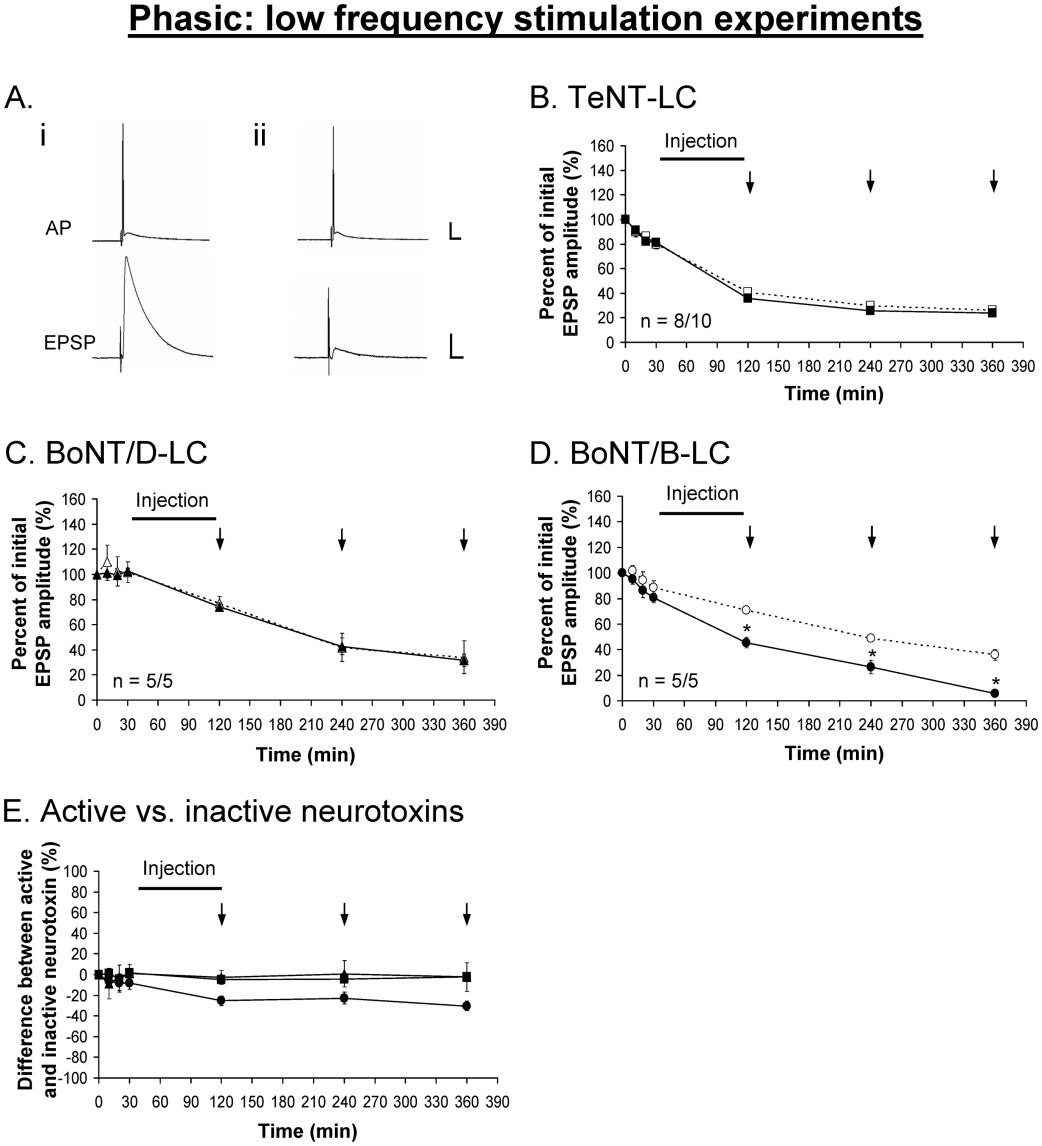
Phasic EPSPs are inhibited using BoNT/B-LC under low frequency stimulation. ***A***, Example showing the phasic action potential (AP) remains unchanged before (*i*) and after (*ii*) the injection of each neurotoxin (BoNT/B-LC used as the example). TeNT-LC (*B*) and BoNT/D-LC (*C*) did not have an effect on the phasic evoked response. ***E***, Percent difference between active and inactive neurotoxin, in which the inactive neurotoxin is the reference point at 0%. In *B-E*, a solid black line represents the time course of neurotoxin injection (90 min) and vertical arrows (↓) represent when EPSPs were recorded. Active neurotoxins: TeNT-LC (▪), BoNT/D-LC (▴) and BoNT/B-LC (•). Inactive neurotoxins: TeNT-LC (□), BoNT/D-LC (△) and BoNT/B-LC (○). Error bars represent S.E.M. An asterisk (*) denotes a significant difference (p<0.05) and ‘n’ represents sample size (active/inactive neurotoxin). Scale bars: horizontal – 10 ms; vertical – 10 mV (AP), 5 mV (EPSP).

**Figure 4:**
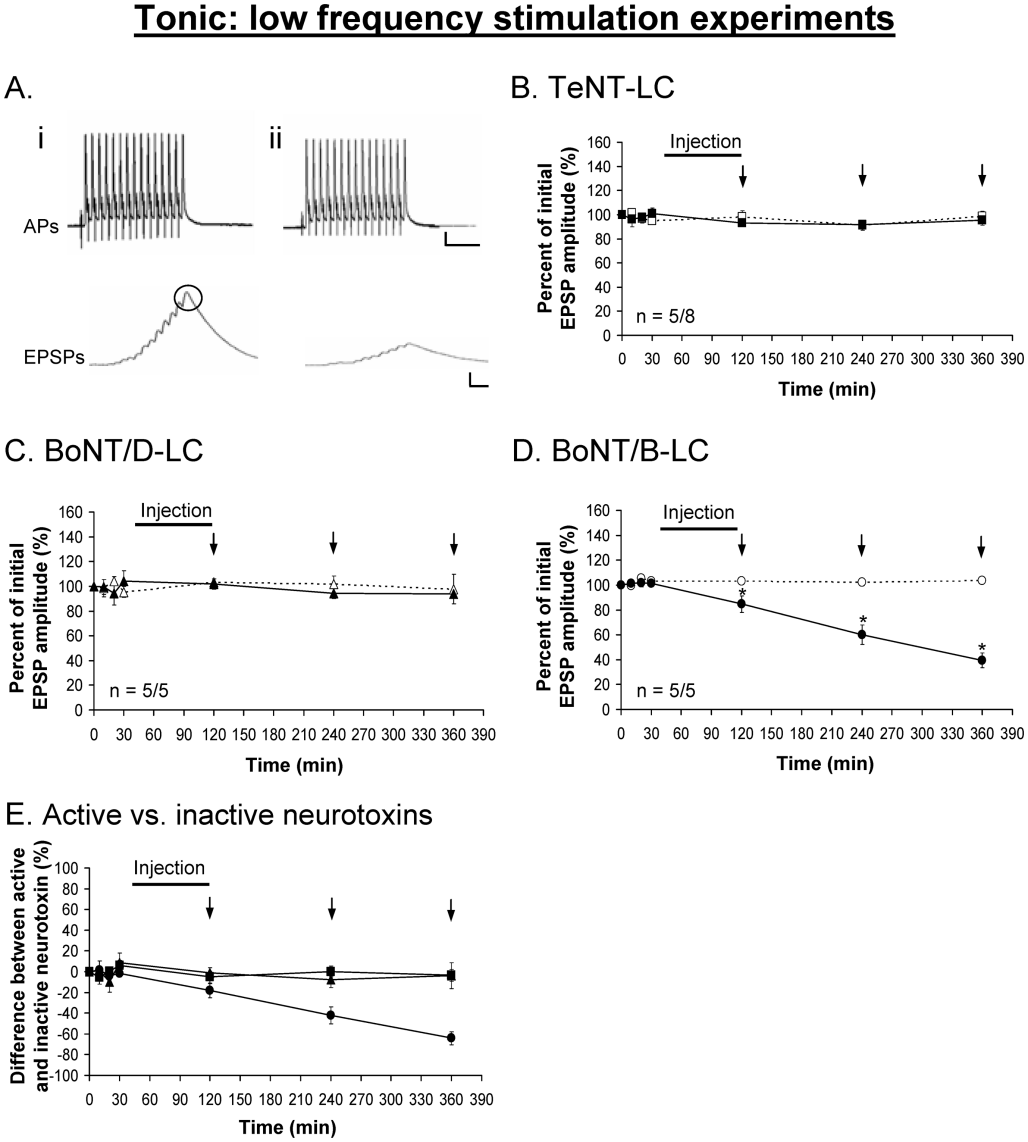
Tonic EPSPs are inhibited using BoNT/B-LC under low frequency stimulation. ***A***, Example showing the tonic action potentials (APs, 200 Hz) remain unchanged before (*i*) and after (*ii*) the injection of each neurotoxin (BoNT/B-LC used as the example). TeNT-LC (*B*) and BoNT/D-LC (*C*) did not have an effect on the tonic evoked response. ***E***, Percent difference between active and inactive neurotoxin, in which the inactive neurotoxin is the reference point at 0%. In *B-E*, a solid black line represents the time course of neurotoxin injection (90 min) and vertical arrows (↓) represent when EPSPs were recorded. Active neurotoxins: TeNT-LC (▪), BoNT/D-LC (▴) and BoNT/B-LC (•). Inactive neurotoxins: TeNT-LC (□), BoNT/D-LC (△) and BoNT/B-LC (○). Error bars represent S.E.M. The measured tonic (last) EPSP is encircled in black (*Ai*). An asterisk (*) denotes a significant difference (p<0.05) and ‘n’ represents the sample size (active/inactive neurotoxin). Scale bars: horizontal – 10 ms; vertical – 10 mV (APs), 5 mV (EPSPs).

**Figure 5:**
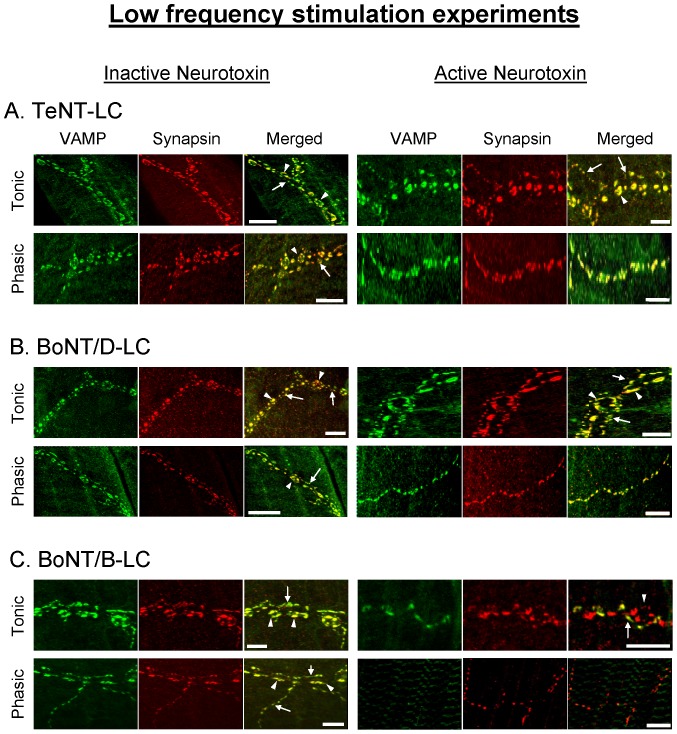
VAMP in phasic and tonic axonal terminals is susceptible only to BoNT/B-LC under low frequency stimulation. Immunostaining of VAMP and synapsin after the injection of inactive and active TeNT-LC (*A*), BoNT/D-LC (*B*) and BoNT/B-LC (*C*) into the phasic or tonic axon during the low frequency stimulation experiments. In *A-C*, arrows denote phasic terminals and arrow heads denote tonic terminals. The yellow areas in the merged image represent an overlap of VAMP and synapsin immunoreactivity. In *C*, the injected boutons contain only synapsin indicating that active BoNT/B-LC cleaved VAMP under low frequency stimulation. Note that only active BoNT/B-LC reduced VAMP immunoreactivity under low frequency stimulation conditions. In *B*,*C*, no tonic terminals were present in the phasic image with active neurotoxin. Scale bars – *A* and *B*: 19 µm; *C*: 10 µm.

The injection of TeNT-LC and BoNT/D-LC had no effect on the evoked phasic (Fig. 3*B,C*) and tonic (Fig. 4*B,C*) responses under the LFS protocol. These results were similar to the effects of the injection of the same but inactivated neurotoxins. Immunocytochemistry performed on the preparations following the physiological experiments showed VAMP staining in both phasic and tonic injected axonal terminals (Fig. 5*A,B*), indicating that VAMP was not cleaved by either neurotoxin. Conversely, the injection of BoNT/B-LC resulted in a significant decline of both the phasic (Fig. 3*D*) and tonic (Fig. 4*D*) evoked responses, first observed immediately after injection. For the remaining 4 hrs of the experiment, however, the tonic response continued to decline in a linear fashion but the phasic response showed no further decline when compared to the control response. Immunocytochemistry showed loss of VAMP staining in injected axonal terminals indicating that VAMP was cleaved (Fig. 5*C*). At the end of the experiment, the inhibitory effect of BoNT/B-LC overall was greater at tonic synapses compared to phasic synapses (65% reduction for tonic (Fig. 4*E*) versus 30% for phasic (Fig. 3*E*), time  =  360 min, p<0.05). The inhibitory effect produced only by BoNT/B-LC indicates that VAMP in both phasic and tonic synapses is associated with *trans-*SNARE complexes that are partially zippered only at the N-terminal end. Therefore, the results indicate that the zippered state of the *trans*-SNARE complex at rest does not solely determine initial release probability.

### Cleavage of VAMP under high frequency stimulation

To verify that partially zippered SNARE complexes can dissociate during repetitive stimulation and to determine whether TeNT-LC and BoNT/D-LC can cleave VAMP in phasic and tonic axons, we applied high frequency stimulation in the presence of the *Clostridial* neurotoxins.

**Figure 6:**
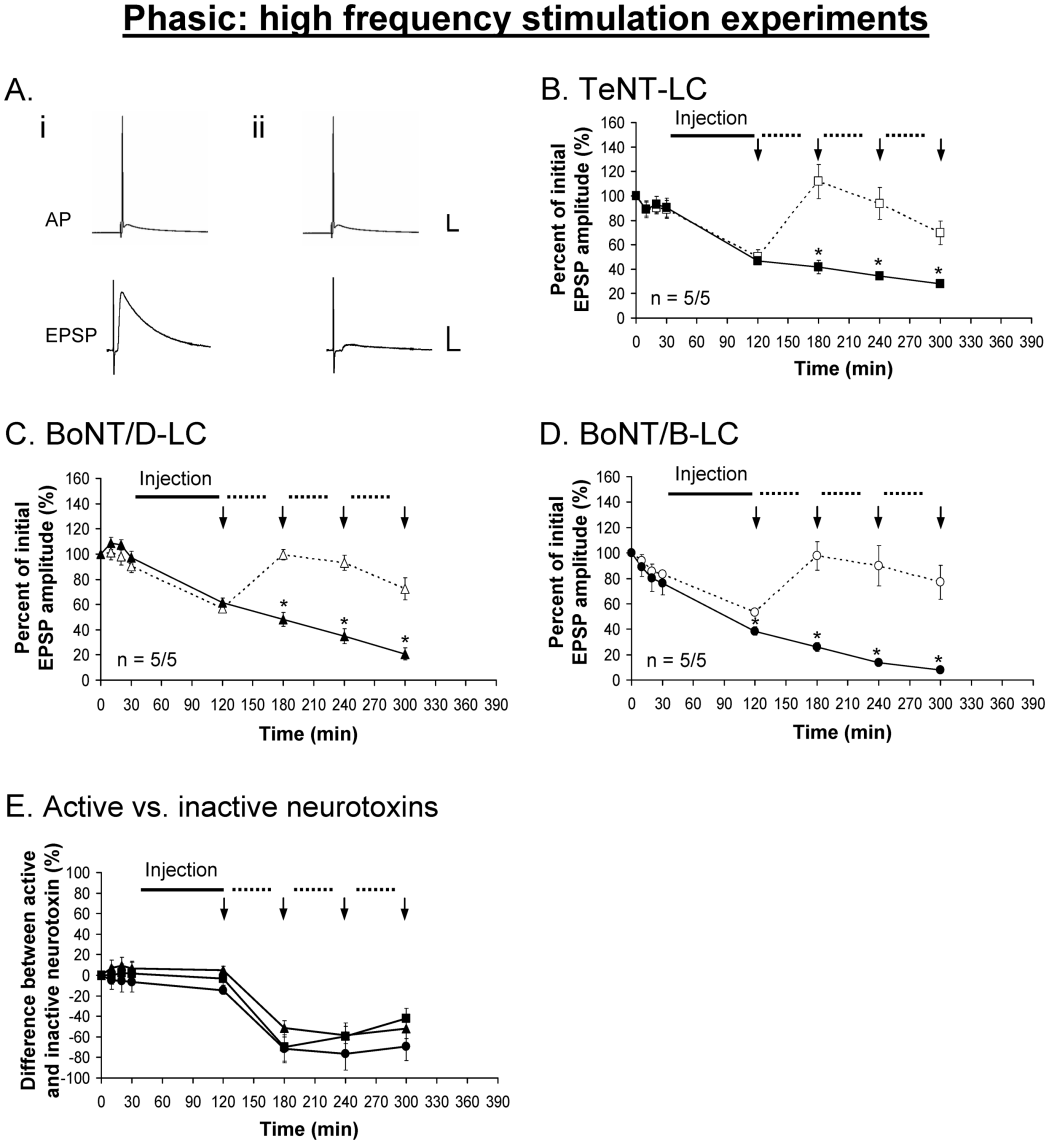
Phasic EPSPs are inhibited using TeNT-LC (*B*), BoNT/D-LC (*C*) and BoNT/B-LC (*D*) under high frequency stimulation. ***A***, Example showing the phasic action potential (AP) remains unchanged before (*i*) and after (*ii*) the injection of each neurotoxin (BoNT/B-LC used as the example). ***E***, Percent difference between active and inactive neurotoxin, in which the inactive neurotoxin is the reference point at 0%. In *B-E,* a solid black line represents the time course of neurotoxin injection (90 min), the dotted lines represent the time course of each round of high frequency stimulation (40 min) and vertical arrows (↓) represent when EPSPs were recorded. Active neurotoxins: TeNT-LC (▪), BoNT/D-LC (▴) and BoNT/B-LC (•). Inactive neurotoxins: TeNT-LC (□), BoNT/D-LC (△) and BoNT/B-LC (○). Error bars represent S.E.M. An asterisk (*) denotes a significant difference (p<0.05) and ‘n’ represents sample size (active/inactive neurotoxin). Scale bars: horizontal – 10 ms; vertical – 10 mV (AP), 5 mV (EPSP).

**Figure 7:**
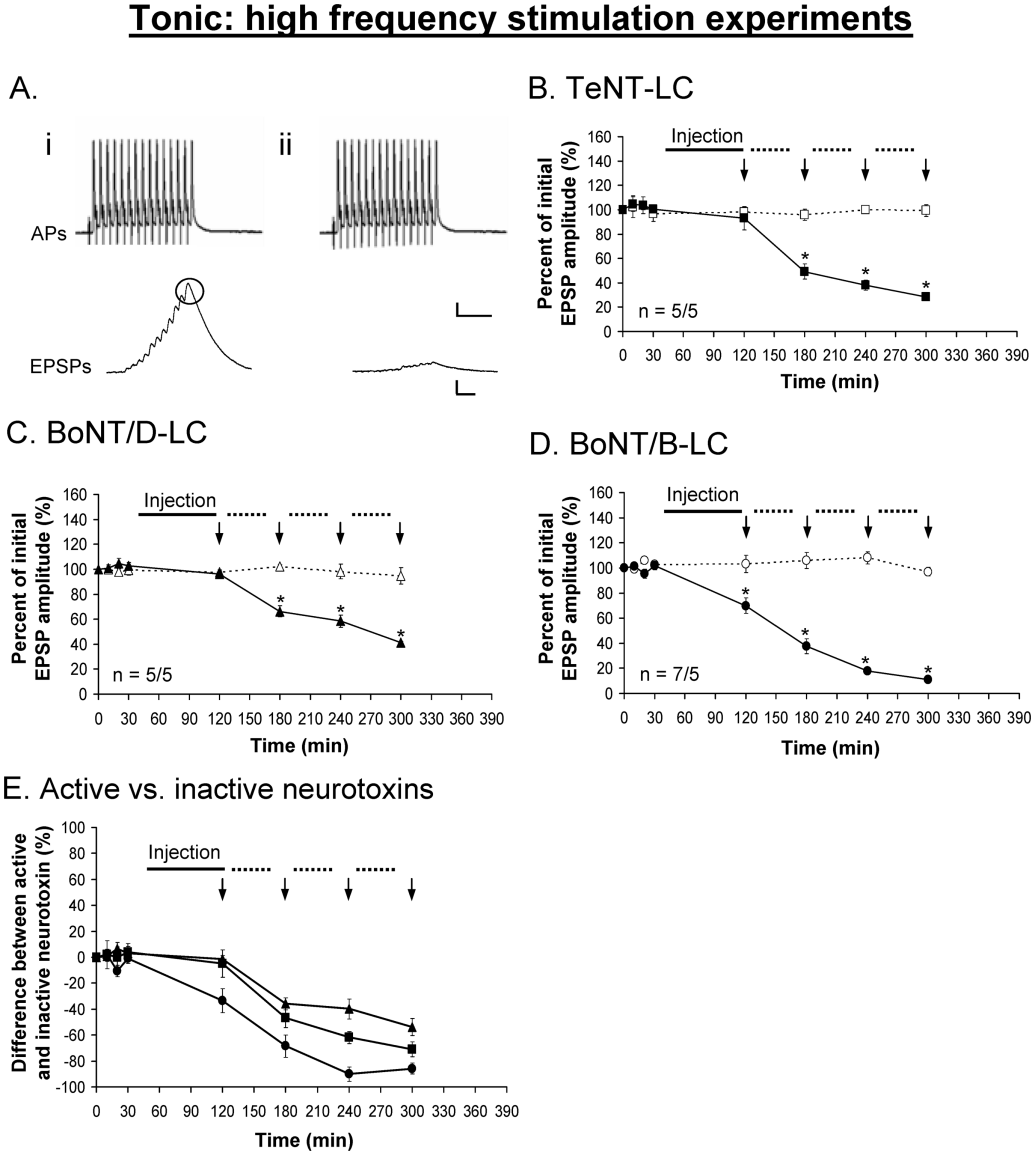
Tonic EPSPs are inhibited using TeNT-LC (*B*), BoNT/D-LC (*C*) and BoNT/B-LC (*D*) under high frequency stimulation. ***A***, Example showing the tonic action potentials (APs) remain unchanged before (*i*) and after (*ii*) the injection of each neurotoxin (BoNT/B-LC used as the example). ***E***, Percent difference between active and inactive neurotoxin, in which the inactive neurotoxin is the reference point at 0%. In *B-E,* a solid black line represents the time course of neurotoxin injection (90 min), the dotted lines represent the time course of each round of high frequency stimulation (40 min) and vertical arrows (↓) represent when EPSPs were recorded. Active neurotoxins: TeNT-LC (▪), BoNT/D-LC (▴) and BoNT/B-LC (•). Inactive neurotoxins: TeNT-LC (□), BoNT/D-LC (△) and BoNT/B-LC (○). Error bars represent S.E.M. The measured tonic (last) EPSP is encircled in black. An asterisk (*) denotes a significant difference (p<0.05) and ‘n’ represents sample size (active/inactive neurotoxin). Scale bars: horizontal – 10 ms; vertical – 10 mV (AP), 5 mV (EPSP).

**Figure 8:**
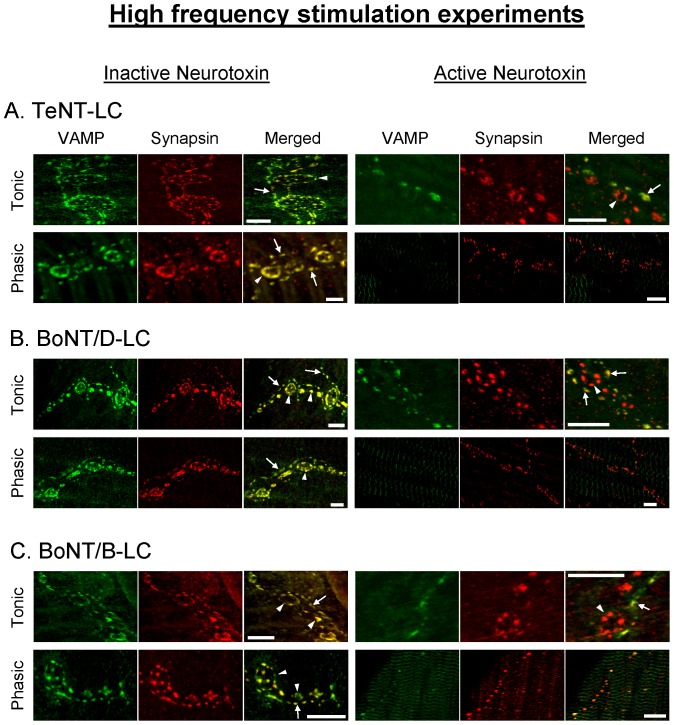
VAMP in phasic and tonic axonal terminals is susceptible to *Clostridial* neurotoxins under high frequency stimulation. Immunostaining of VAMP and synapsin following the high frequency stimulation experiments using inactive and active TeNT-LC (*A*), BoNT/D-LC (*B*) and BoNT/B-LC (*C*). In *A-C*, arrows denote phasic terminals and arrow heads denote tonic terminals. Areas of overlap between VAMP and synapsin immunoreactivity are denoted in yellow in the merged image. Note that all three active neurotoxins reduced VAMP immunoreactivity. In *A*-*C*, no tonic terminals were present in the phasic image with active neurotoxin. Scale bars: 10 µm.

The injection of TeNT-LC, BoNT/B-LC and BoNT/D-LC under high frequency stimulation resulted in a significant decrease of the evoked phasic (Fig. 6) and tonic (Fig. 7) responses. Both phasic and tonic responses showed a significant decline immediately after injection of BoNT/B-LC but not with BoNT/D-LC and TeNT-LC prior to high frequency stimulation. This was similar to the results using the LFS protocol, again demonstrating that VAMP is at least partially exposed in the *trans*-SNARE complex and accessible only to BoNT/B-LC under resting conditions. The inhibitory effects of TeNT-LC and BoNT/D-LC were initially observed following the first round of high frequency stimulation at both phasic and tonic synapses (Figs. 6*B,C*, 7*B,C*, time = 180 min). The responses showed the greatest inhibition after the first round of intense stimulation and thereafter continued to decline over time but at a slower rate. Overall, BoNT/B-LC had the greatest inhibitory effect over time for both phasic and tonic responses, accelerated by stimulation (∼80% reduction), followed by BoNT/D-LC and TeNT-LC, both of which elicited a similar amount of decline over time (peak reduction of ∼60–70%, see Figs. 6*E*, 7*E*). In addition, the three active neurotoxins eliminated the potentiated phasic response normally observed during intense stimulation for the controls. This suggests that VAMP was cleaved not only on vesicles in the RRP but also in the reserve pool, which would be recruited during intense stimulation. Immunocytochemistry performed on the preparations following the physiological experiments (different from the ones used for the LFS experiments) showed no VAMP staining (VAMP was cleaved) when each active neurotoxin inhibited the phasic and tonic responses (evident only when stimulation was applied using TeNT-LC and BoNT/D-LC, see Fig. 8). In comparison to the results of the LFS experiments, all three neurotoxins had a greater inhibitory effect under high frequency stimulation at both phasic and tonic synapses (p<0.05, time = 240 min, Figs. 3*E* *vs.* 6*E* (phasic) and 4*E* vs. 7*E* (tonic)). In addition, action potentials recorded from the phasic and tonic axons under all conditions using all three neurotoxins were unaffected (see Figs. 3*A*, 4*A*, 6*A*, 7*A*). Therefore, the action of BoNT/B-LC at phasic and tonic synapses proceeded with minimal stimulation, although the effect was facilitated with stimulation, whereas the action of TeNT-LC and BoNT/D-LC were stimulation-dependent.

## Discussion

### Partially zippered *trans*-SNARE complexes at phasic and tonic synapses

This is the first test of the hypothesis that, in the resting state, SNARE zippering past the zero-layer of the *trans*-SNARE complex is associated with a higher probability of NT release. The effects of BoNT/B-LC, BoNT/D-LC and TeNT-LC under low and high frequency stimulation were similar at both phasic and tonic synapses. The inhibitory effect of each neurotoxin was accompanied by cleavage of VAMP in the axonal terminals as shown by the reduction of immunoreactivity. This observation demonstrates the specificity of the neurotoxins. The loss of VAMP immunoreactivity did not correspond quantitatively with the loss of NT release probably because most of the immunoreactivity is on vesicles that are at a distance from the active zone. It is probably only necessary to cleave VAMP on docked vesicles to block NT release. This is similar to our observations at the frog neuromuscular junction, where application of BoNT/D holotoxin produced complete block of NT release in 1.5 hours but 43% of the VAMP immunoreactivity remained [40].

The cleavage of VAMP separates its transmembrane domain from its cytosolic domain. This will still allow the cytosolic domain containing the SNARE binding motif to interact with the t-SNAREs (syntaxin and SNAP-25) but form a *cis*-SNARE complex [15], [17] that is not fusogenic, resulting in the failure of vesicle fusion. Therefore, as more VAMP proteins are cleaved, the inhibitory effect of the neurotoxins increases. This would explain why BoNT/B-LC produced a greater inhibitory effect compared to the other two neurotoxins since BoNT/B-LC does not require high synaptic activity to cleave VAMP; BoNT/B-LC can cleave VAMP during injection at rest and thus evoke an inhibitory effect earlier than the other neurotoxins during the course of the experiment.

**Figure 9:**
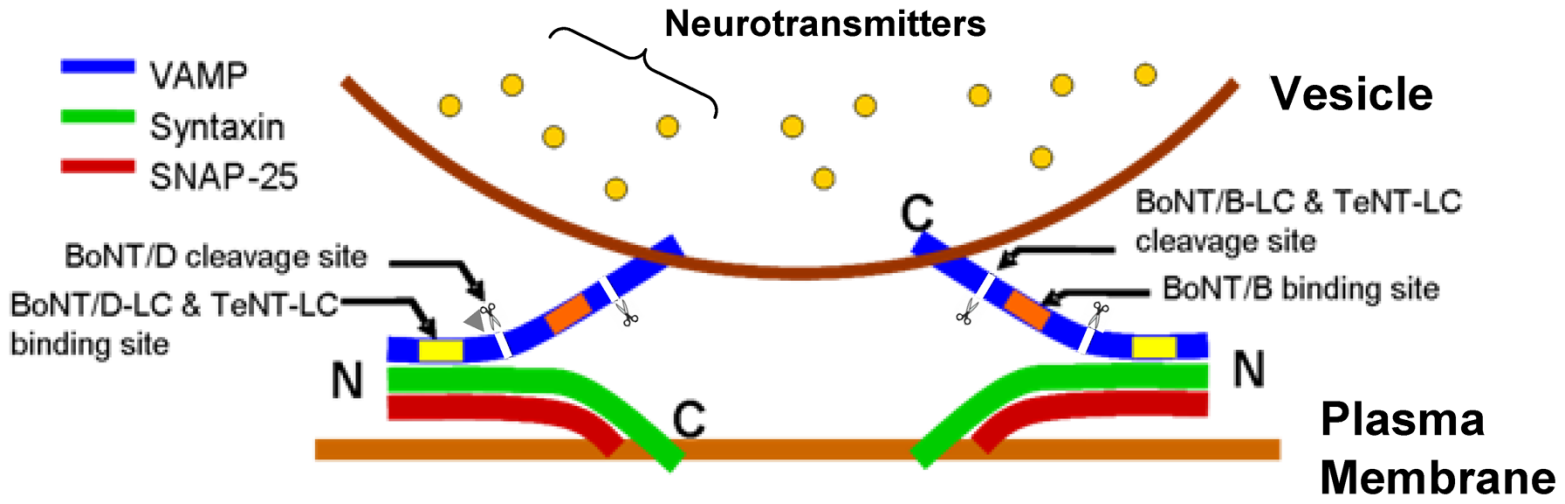
The *trans*-SNARE complexes at phasic and tonic synapses are partially zippered under resting state. The N-terminal end of the SNARE complex is zippered whereas the C-terminal end is exposed. The binding and cleavage sites of BoNT/B are exposed but the shared binding site for TeNT and BoNT/D, and possibly the cleavage site for BoNT/D, are occluded. Scissors and white lines indicate neurotoxin cleavage site. Gray arrow head indicates the location of the zero-layer across all three SNARE proteins.

The effects of the three neurotoxins under low frequency stimulation can be explained by the existence of partially zippered *trans*-SNARE complexes. The data suggest that *trans*-SNARE complexes are partially zippered from the N-terminal end to approximately the zero-layer of the SNARE binding motif of each SNARE (Fig. 9). In this state, the binding site common to both BoNT/D and TeNT (N-terminal region of VAMP's SNARE motif), and possibly the cleavage site of BoNT/D, are occluded whereas the cleavage site common to both BoNT/B and TeNT, and the binding site of BoNT/B are exposed C-terminal to the zero-layer. Therefore, only BoNT/B can bind to and cleave VAMP in the resting state, which was observed in our experiments. The finding that BoNT/B-LC inhibited release at both phasic and tonic synapses under resting state falsified our original hypothesis of *trans*-SNARE complexes zippered past the zero-layer are exclusively required at high release probability synapses at rest. This led us to conclude that initial release probability is not determined solely on the zippered state of the *trans*-SNARE complex.

In the Western blot experiment (Fig. 1*B*), unlike the low stimulation results, VAMP was cleaved by all three neurotoxins. In those experiments, the nerve cord protein had been extracted in denaturing conditions and subsequently boiled and cooled. That procedure allowed the protein to retain its tertiary structure but disrupted interactions with other proteins prior to subjecting the protein sample to proteolysis and gel electrophoresis. Therefore, VAMP was susceptible to cleavage by the three neurotoxins because the binding and cleavage sites of each neurotoxin were exposed on the protein.

The use of both low and high frequency stimulation helped to distinguish the effects of the neurotoxins under resting and non-resting states. For instance, the LFS protocol was designed to evoke minimum synaptic activity to study the zippered state of the SNARE complex under resting state, which cannot be determined with high frequency stimulation due to rapid turnover of SNAREs. In addition, the low frequency of spontaneous release from the crayfish phasic and tonic terminals (∼2–4 quanta/min, [21], [41]) reflects a low rate of turnover of the SNAREs, further contributing to the low level of activity under resting state. In secretory systems in which there is a high frequency of spontaneous release, rapid turnover of SNARE complexes or few docked vesicles, the effects of TeNT-LC, BoNT/B-LC and BoNT/D-LC might not be different. We also observed that under conditions in which the neurotoxins had an inhibitory effect the synaptic response was not abolished as found by [6], [20]. Immunocytochemistry showed loss of VAMP immunoreactivity under conditions that resulted in a decrease of evoked release using active neurotoxins; however, the presence of small evoked responses indicated that a small fraction of VAMP proteins were not cleaved by the active neurotoxins. Unfortunately, our confocal microscope could not identify this small pool of uncleaved VAMP. Evidently, the neurotoxins did not provide complete proteolysis of all VAMP proteins at both synapses during the time course of the LFS and HFS experiments. Nevertheless, there were large differences in the stimulus-dependent effects of the neurotoxins.

It is unclear under resting state if *trans*-SNARE complexes fluctuate between different zippered states as observed in slower exocytotic systems [9], [13]. The results of BoNT/B-LC under the LFS protocol would be in favour of this model: *trans*-SNARE complexes at tonic synapses fluctuate between different states, in which VAMP is more frequently exposed, whereas a greater fraction exists in a more tightly zippered state at phasic synapses. This could explain why BoNT/B-LC was more effective at tonic synapses than at phasic synapses. In this model, however, *trans*-SNARE complexes cannot unzipper N-terminal to the shared binding site of TeNT and BoNT/D-LC under resting state, otherwise TeNT and BoNT/D-LC would produce a similar inhibitory effect as BoNT/B-LC under the LFS protocol, which was not observed. Unfortunately, our experiments cannot determine if there are small fluctuations in the zippered state of the *trans*-SNARE complex. It is possible that a difference in the proportion of *trans*-SNARE complexes that exhibit small fluctuations in their zippered state could contribute to the difference in release probability between the phasic and tonic synapses: a greater ratio of zippered to unzippered complexes would yield a higher release probability.

A previous study by [42] showed that the SNARE complex forms in 3 steps and pauses at the zero-layer residue R56 (rat VAMP2) leaving the binding and cleavage sites for BoNT/B-LC exposed. When the N- and C-terminal domains have zippered, the linker domains form a low energy complex. Formation of the linker domain complex might represent another site for control of NT release but this could not be tested with the present techniques.

### Differential effects of BoNT/B-LC

This study used the indirect approach of identifying the zippered state of the SNARE complex based on whether or not BoNT/B-LC, BoNT/D-LC and TeNT-LC produced an inhibitory effect, and therefore cleaved VAMP, under resting conditions. The assessment of the zippered state is not dependent on the degree of inhibition but rather on the ability of each neurotoxin to produce an inhibitory effect under the LFS paradigm. The observation that BoNT/B-LC, but not BoNT/D-LC and TeNT-LC, inhibited phasic and tonic responses under LFS was sufficient to conclude that the *trans*-SNARE complex was partially zippered from the N-terminal end to approximately the zero-layer at both synapses. If *trans*-SNARE complexes were tightly zippered beyond the zero-layer at phasic synapses then BoNT/B-LC would not have produced an inhibitory effect under the LFS paradigm because, at the very least, the binding site of BoNT/B-LC would be occluded. Therefore, BoNT/B-LC would have yielded a similar effect as BoNT/D-LC and TeNT-LC under LFS and HFS, which was not observed in this study.

There is likely more than one explanation for the different inhibitory effects produced by BoNT/B-LC but our experiments could not investigate this phenomenon in greater detail. We speculate that there are fewer *trans*-SNARE complexes at docked vesicles of tonic synapses compared to phasic synapses, and therefore cleavage of VAMP could more easily reduce intact VAMP to a critical level insufficient to support NT release [43]. Future experiments that can provide more accurate information about the number of partially zippered *trans*-SNARE complexes, the degree of zippering and the number of free VAMP proteins at phasic and tonic synapses will be needed to address the differential effect of BoNT/B-LC observed in this study.
